# Self-Managed Non-Pharmacological Interventions for Breast Cancer Survivors: Systematic Quality Appraisal and Content Analysis of Clinical Practice Guidelines

**DOI:** 10.3389/fonc.2022.866284

**Published:** 2022-05-30

**Authors:** Jing-Yu (Benjamin) Tan, Jianxia Zhai, Tao Wang, Hong-Juan Zhou, Isabella Zhao, Xian-Liang Liu

**Affiliations:** ^1^ Charles Darwin University, College of Nursing and Midwifery, Casuarina, NT, Australia; ^2^ Charles Darwin University, College of Nursing and Midwifery, Melbourne Hub, Melbourne, VIC, Australia; ^3^ Charles Darwin University, College of Nursing and Midwifery, Brisbane Centre, Brisbane, QLD, Australia; ^4^ Fujian University of Traditional Chinese Medicine, School of Nursing, Minhou, China; ^5^ Queensland University of Technology, Cancer and Palliative Care Outcomes Centre, Brisbane, QLD, Australia

**Keywords:** breast cancer, self-management, non-pharmacological interventions, clinical practice guidelines, content analysis

## Abstract

**Background:**

A growing number of clinical practice guidelines (CPGs) regarding non-pharmacological interventions for breast cancer survivors are available. However, given the limitations in guideline development methodologies and inconsistent recommendations, it remains uncertain how best to design and implement non-pharmacological strategies to tailor interventions for breast cancer survivors with varied health conditions, healthcare needs, and preferences.

**Aim:**

To critically appraise and summarise available non-pharmacological interventions for symptom management and health promotion that can be self-managed by breast cancer survivors based on the recommendations of the CPGs.

**Methods:**

CPGs, which were published between January 2016 and September 2021 and described non-pharmacological interventions for breast cancer survivors, were systematically searched in six electronic databases, nine relevant guideline databases, and five cancer care society websites. The quality of the included CPGs was assessed by four evaluators using The Appraisal of Guidelines for Research and Evaluation, second edition tool. Content analysis was conducted to synthesise the characteristics of the non-pharmacological interventions recommended by the included CPGs, such as the intervention’s form, duration and frequency, level of evidence, grade of recommendation, and source of evidence.

**Results:**

A total of 14 CPGs were included. Among which, only five were appraised as high quality. The “range and purpose” domain had the highest standardized percentage (84.61%), while the domain of “applicability” had the lowest (51.04%). Five CPGs were rated “recommended”, seven were “recommended with modifications”, and the other two were rated “not recommended”. The content analysis findings summarised some commonly recommended self-managed non-pharmacological interventions in the 14 guidelines, including physical activity/exercise, meditation, hypnosis, yoga, music therapy, stress management, relaxation, massage and acupressure. Physical activity/exercise was the most frequently recommended approach to managing psychological and physical symptoms by the included guidelines. However, significant variations in the level of evidence and grade of recommendation were identified among the included CPGs.

**Conclusion:**

Recommendations for the self-managed non-pharmacological interventions were varied and limited among the 14 CPGs, and some were based on medium- and low-quality evidence. More rigorous methods are required to develop high-quality CPGs to guide clinicians in offering high-quality and tailored breast cancer survivorship care.

## 1 Introduction

Approximately 2.3 million women were diagnosed with breast cancer worldwide in 2020, and breast cancer resulted in more lost disability-adjusted life years than other types of cancer around the world (1). Breast cancer poses an extensive threat to women’s physical and psychological well-being globally (2). Advances in treatments have contributed to improved survival globally (3); in particular, the average five-year survival rate for women with non-metastatic breast cancer reached 96% in Australia (4). Newer treatments and interventions have shifted breast cancer from a fatal illness to a chronic condition, which has resulted in more breast cancer survivors living with persistent symptoms, such as nausea, vomiting, fatigue, pain, and sleep disturbance (5). Breast cancer survivors’ quality of life (QoL) can be negatively impacted by these distressing symptoms, which should be addressed by multidisciplinary healthcare professionals throughout the breast cancer trajectory (6).

To deal with the multitude of distressing symptoms, breast cancer survivors often explore different approaches, ranging from pharmacological to non-pharmacological modalities. Due to some potential unpleasant reactions, such as nausea, vomiting, skin reactions, headaches, and drug-drug interactions, in conventional pharmacological treatment (7), it is necessary to explore safe and effective nonpharmacological interventions for individuals with breast cancer. In addition, due to the current oncologist-led model of care that substantially emphasises detecting recurrences, there is a lack of sufficient support from healthcare professionals to manage unpleasant long-term physical and psychological symptoms in breast cancer survivors during the follow-up period (8). As a result, it can be challenging to meet breast cancer survivors’ comprehensive physical, psychological, and social needs (9, 10). A body of evidence showed that some effective non-pharmacological strategies might positively improve functional outcomes and QoL in breast cancer survivors (11–13). There has been a shift towards self-management, which has been proposed as a strategy to address breast cancer survivors’ long-term health needs physically and psychologically (14). Self-management refers to patients’ ability, with or without the support of their family and community and along with the oversight of clinicians, to handle the psychosocial and physical aspects of their chronic conditions (15, 16). Self-management strategies are an essential part of cancer survivorship care as they can enhance survivors’ self-efficacy and empower them to manage their conditions, thereby sustaining a satisfactory QoL (17). A large body of evidence has demonstrated that self-management approaches have the potential to enhance a wide range of physical and psychosocial outcomes (e.g., fatigue, psychological distress, sleep disturbance, etc.) and reduce healthcare use among individuals with chronic conditions (18, 19), including breast cancer (16).

Clinical practice guidelines (CPGs) are evidence-based reference documents, including recommendations for diagnosis and treatment and care of people with particular types of disease, which can help end-users promote clinical practices (20). The use of oncology CPGs has been demonstrated to enhance the overall survival and management of cancer (21, 22). Although efforts to integrate the evidence have resulted in the development of several CPGs pertaining to self-managed non-pharmacological interventions for breast cancer survivors (23–27), the CPGs used their own specific methodologies for guideline development and evidence grading, emphasising specific breast cancer samples and stages and particular types of clinical outcomes, which contributed to inconsistent recommendations across CPGs (23–30), and might further hinder clinicians in decision-making as well as guiding best practices in breast cancer management.

To the best of our knowledge, no previous systematic appraisals of CPGs for self-managed non-pharmacological interventions in breast cancer survivors have been conducted. In response to the growing calls for the promotion of self-management for breast cancer survivors as well as the limitations of recommendations in the current CPGs on this topic, a review of CPGs exploring self-managed non-pharmacological interventions for breast cancer survivors was conducted to summarise the best available evidence.

Specifically, the objectives of this review were (1): to critically appraise the quality of the analysed CPGs (2); to identify the level of evidence and degree of recommendation for each non-pharmacological self-management intervention; and (3) to summarise and analyse the contents of the available non-pharmacological interventions that can be self-managed by breast cancer survivors.

## 2 Methods

A structured umbrella review was carried out in line with the methodology suggested by Smith, Devane (31). The Preferred Reporting Items for Systematic Reviews and Meta‐Analyses (PRISMA) checklist (32) was adopted to guide this review. This review has been registered at INPLASY(INPLASY.COM). The registered number is INPLASY202230175 (doi: 10.37766/inplasy2022.3.0175). A pre-print version of this manuscript is also available at https://www.preprints.org/manuscript/202203.0102/v1.

### 2.1 Search Strategy

A comprehensive search of literature was performed in September 2021 to identify eligible guidelines published during the last five years (1): six online databases, including PubMed, Cochrane Library, Medline, PsycINFO, Web of Science, and CINAHL (2): nine guideline repositories, including the Guideline International Network, the National Guideline Clearinghouse, the Scottish Intercollegiate Guidelines Network, the National Comprehensive Cancer Network, the National Institute for Health and Care Excellence, the Australian Clinical Practice Guidelines Portal, the New Zealand Guidelines Group, the Canadian Medical Association Infobase, and the Trip Medical Database; and (3) official websites from five professional cancer associations, including the Multinational Association of Supportive Care in Cancer, Cancer Council Australia, the Oncology Nursing Society (ONS), the American Cancer Society (ACS), and the American Society of Clinical Oncology (ASCO). A representative search strategy in PubMed is presented in **Table 1**.

**Table 1:** Search strategy in PubMed.

#1	breast neoplasm [MeSH Terms]
#2	(((((((((((((Breast Neoplasm*[Title/Abstract]) OR (Breast tumor*[Title/Abstract])) OR (Breast cancer*[Title/Abstract])) OR (Breast carcinoma*[Title/Abstract])) OR (Mammary cancer*[Title/Abstract])) OR (Mammary carcinoma*[Title/Abstract])) OR (Mammary neoplasm*[Title/Abstract])) OR (Mammary tumor*[Title/Abstract])) OR (Malignant neoplasm of breast[Title/Abstract])) OR (Breast malignant neoplasm*[Title/Abstract]))) OR (Malignant tumor of breast[Title/Abstract])) OR (Breast malignant tumor*[Title/Abstract])) OR (Cancer of breast[Title/Abstract])
#3	#1 OR #2
#4	guideline [MeSH Terms]
#5	((((((((((guideline[Publication Type]) OR (Practice Guideline[Publication Type]))) OR (guideline*[Title/Abstract])) OR (Best Practice*[Title/Abstract])) OR (Recommendation*[Title/Abstract])) OR (Consensus*[Title/Abstract])) OR (Experts Opinion*[Title/Abstract])
#6	#4 OR #5
#7	#3 AND #6

### 2.2 Inclusion and Exclusion Criteria

The included CPGs met the following criteria (1): CPGs that were published in refereed English academic journals, collected guideline databases, or published by relevant professional cancer associations in the last five years (since January 2016) (2): CPGs that focused on breast cancer survivors, regardless of the stages of diagnosis and types of antineoplastic therapies (3): CPGs that presented any type of non-pharmacological strategies that can be self-managed by breast cancer survivors regardless of the types of delivery method and format, such as yoga, physical exercise, music therapy, meditation, massage, relaxation, acupressure, etc (4): when there are multiple editions of a CPG, only the latest version was included (5): when there are different language/translation versions of a CPG, only the English version was included. CPGs were excluded if they (1): included pharmacological or surgical interventions only (2): were patient-used guidelines, which offer evidence-based recommendations in general without providing detailed evidence analysis, auditing criteria, grade of recommendation, etc.

### 2.3 Study Selection and Data Extraction

Duplications were identified and removed *via* the literature management software EndNote X9. Two independent reviewers (JZ and TW) read the titles and abstracts of the remaining CPGs to select and analyse those that could be potentially included. Then, full-text reviews of the potentially eligible CPGs were conducted by the same two reviewers. Eligible CPGs were eventually included based upon the inclusion and exclusion criteria. Key information in each CPG was extracted using predefined tables, including (1): the characteristics of the included guidelines, such as the name of the CPG, developer, year of publication, whether publication was in a journal, evidence analysis, quality tool referral, etc.; and (2) the contents of the non-pharmacological interventions that were recommended by the included guidelines, such as the form, duration and frequency, level of evidence (LoE), source of evidence (SoE), and grade of recommendation (GoR). In any case of disagreement, a team meeting was organised to resolve the issues during the data retrieval and extraction process.

### 2.4 Quality Assessment

To critically assess the quality of the included guidelines, the Appraisal of Guidelines for Research and Evaluation, 2nd Edition (AGREE II) was utilised. The AGREE II consists of 23 items, which can be utilised to evaluate the quality of CPGs’ development, transparency, and methodological rigor in six domains: “scope and purpose”, “stakeholder involvement”, “rigor of development”, “clarity and presentation”, “applicability”, and “editorial independence” (33). The AGREE II uses a 7-point Likert scale, from 1 (strongly disagree) to 7 (strongly agree), to assess each item (33). For the global quality and level of the recommendations, a CPG would be considered a grade of “recommended” (high quality) when the mean percentages of the six standardised domains was greater than 70%, a grade of “recommended with modifications” (moderate quality) when the standardised percentages were between 40% and 70% in over three domains, and a grade of “not recommended” (low quality) once the standardised percentages were lower than 40% in three domains or more (34). Four independent assessors critically appraise the quality of each included CPG. All four assessors were experienced academics and health practitioners with over 10 years of professional experience in evidence-based practice, cancer research, and CPG quality appraisal. Each assessor participated the AGREE II Overview Tutorial and the online AGREE II Tutorial and Practice Exercise (33) to effectively apply the instrument to GPG quality appraisal.

### 2.5 Data Analysis

Consistency among the assessors in the quality assessment of the CPGs was examined using the intraclass correlation coefficient (ICC): ICC of 0.75 or greater suggests satisfactory consistency as per the recommendations (35). The statistical analyses for the ICC were conducted using the SPSS 25. A value of *p* < 0.05 indicated statistical significance. Content analysis (36) was adopted to summarise and categorise the self-managed non-pharmacological approaches in the 14 guidelines. Symptoms (e.g., anxiety/depression, fatigue, pain, etc.), quality of life, and risk of recurrence were predetermined themes for the content analysis based on the aims and scopes of the included CPGs that made recommendations on a range of clinical outcomes. the “health promotion” theme was further added after multiple iterative, deductive, and inductive processes (36).

## 3 Results

**Figure 1:**
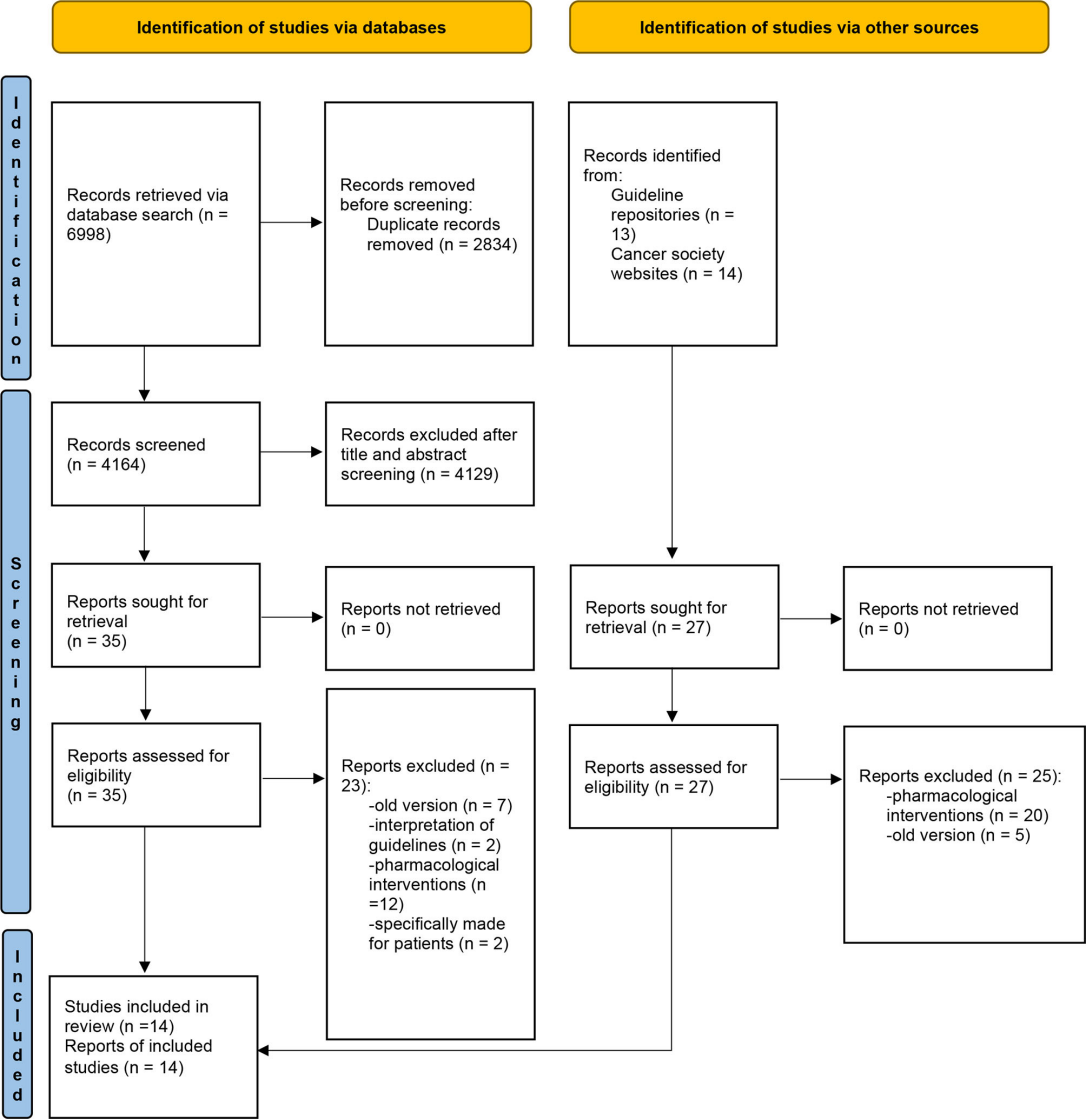
PRISMA flow diagram of study selection. *Adapted from:* Page MJ, McKenzie JE, Bossuyt PM, Boutron I, Hoffmann TC, Mulrow CD, et al. The PRISMA 2020 statement: an updated guideline for reporting systematic reviews. BMJ 2021;372.

The literature search in the databases generated 6,998 results, while the guideline repository and professional cancer care website searches yielded 27 results. In total, 7,025 records were located, 2,834 of which were removed for duplication. After screening the titles and abstracts, 4,129 records were further excluded. The remaining 62 full-text records were reviewed for eligibility. A further 48 records were excluded, which led to the final inclusion of 14 guidelines **(** **Figure 1** **)**.

### 3.1 Characteristics of the Included Clinical Practice Guidelines

All included CPGs (26–30, 37–45) were published or updated between 2016 and 2021 **(** **Table 2** **)**, of which four were from Europe, four from the US, two from the UK, two from Spain, and one each from German and Canada. Eight guidelines (57.14%) were updated versions, while the rest were newly developed. Most the guidelines (12/14, 85.71%) were specifically designed for breast cancer survivors, while the other two also focused on prostate cancer and colorectal cancer. Twelve of the 14 (85.71%) guidelines were published in a journal, while two guidelines were published on the NICE website (28, 42).

**Table 2:** Characteristics of the included clinical practice guidelines.

Name of CPG	Abbreviated Name	Developer	Year Published/Updated	Publication in a Journal	Newly Developed	Continent/country	Evidence Analysis	Quality Tool Referral
Advanced breast cancer: diagnosis and treatment (CG81) (42)	NICE (CG81)	NICE	2017	Not published	No	UK	Systematic review; consensus method among experts, including patient/carer	AGREE II
Early and locally advanced breast cancer: diagnosis and management (NG101) (28)	NICE (NG101)	NICE	2018	Not published	No	UK	Systematic review; consensus method among experts, including patient/carer	AGREE II
Recommendations for the follow-up care of female breast cancer survivors (37)	Barnadas, Algara (37)	SEOM, SEMERGEN, SEMFYC, SEMG, SEGO, SEOR, SESPM, SEC	2018	Clinical and Translational Oncology	Yes	Spain	Consensus method, not specified	Not reported
Estimating the benefits of therapy for early-stage breast cancer: the St. Gallen International Consensus Guidelines for the primary therapy of early breast cancer 2019 (38)	St. Gallen International Consensus	St. Gallen	2019	Annals of Oncology	No	Europe	Nominal group technique	Not reported
SEOM clinical guidelines in early-stage breast cancer (27)	SEOM guideline	SEOM	2018	Clinical and Translational Oncology	No	Spain	Consensus method, not specified	Not reported
Early breast cancer: ESMO Clinical Practice Guidelines for diagnosis, treatment, and follow-up (39)	ESMO (EBC)	ESMO	2019	Annals of Oncology	No	Europe	Review	Not reported
ESO-ESMO 4th International Consensus Guidelines for Breast Cancer in Young Women (BCY4) (43)	ESO-ESMO (BCY4)	ESO, ESMO	2020	Annals of Oncology	No	Europe	Consensus method, expert panel review, including patient advocates	Not reported
5th ESO-ESMO International Consensus Guidelines for Advanced Breast Cancer (ABC 5) (40)	ESO-ESMO (ABC 5)	ESO, ESMO	2020	Annals of Oncology	No	Europe	Nominal group technique, including patient advocates	Not reported
Interventions for Breast Cancer-Related Lymphedema: Clinical Practice Guideline from the Academy of Oncologic Physical Therapy of APTA (41)	APTA guideline	APTA	2020	Physical Therapy	Yes	US	Review	Not reported
Clinical Practice Guidelines on the Evidence-based Use of Integrative Therapies During and After Breast Cancer Treatment (30)	SIO guideline	SIO	2017	CA: A Cancer Journal for Clinicians	No	US	Systematic review	Not reported
ONS Guidelines™ for Cancer Treatment-related Hot Flashes in Women With Breast Cancer and Men With Prostate Cancer (26)	ONS guideline	ONS	2020	Oncology Nursing Forum	Yes	US	Systematic review, consensus method among experts, including patient representative	Not reported
Practice guidelines for psychological interventions in the rehabilitation of patients with oncological disease (breast, prostate, or colorectal cancer) (44)	Reese, Weis (44)	University of Freiburg	2016	Psycho‐Oncology	Yes	Germany	Review, consensus method (expert panel), including patients (focus group)	Not reported
American Cancer Society (ACS)/American Society of Clinical Oncology (ASCO) Breast Cancer Survivorship Care Guideline (45)	ACS-ASCO guideline	ACS, ASCO	2016	CA: A Cancer Journal for Clinicians	Yes	USA	Systematic review, consensus method (expert workshop), including a patient	Not reported
Follow-up after treatment for breast cancer: Practical guide to survivorship care for family physicians (29)	Sisler, Chaput (29)	College of Family Physicians	2016	Canadian Family Physician	Yes	Canada	Review	Not reported

NICE, National Institute for Health and Care Excellence; UK, United Kingdom; AGREE II, Appraisal of Guidelines for Research and Evaluation, second edition; SEOM, Sociodad Española de Oncolgía Médica; ESMO, European Society for Medical Oncology; EBC, early breast cancer; ESO, European School of Oncology; ABC, advanced breast cancer; APTA, American Physical Therapy Association; US, United States; SIO=Society of Interventional Oncology; ONS, Oncology Nursing Society.

Half of the included CPGs engaged with cancer survivors as the key stakeholder during the guideline development. A systematic review approach was adopted in only four CPGs as part of the development methodology with comprehensive database search, specific inclusion criteria, data selection and synthesis. Only two guidelines (NICE guidelines) adopted the AGREE II tool to inform the guideline development process (28, 42).

### 3.2 Methodological Quality of the Included Clinical Practice Guidelines

The ICC between the four reviewers for each guideline ranged from 0.956 to 0.995, with an average of 0.980, indicating a high consistency of rating scores among the four reviewers. According to the AGREE II instrument, the mean overall standardised percentage of the guidelines was 67.49%, ranging from 36.12% to 93.82% **(** **Table 2** **)**. The following five guidelines were rated as high quality, with an indication of recommended for use: the NICE (CG81) guideline, the NICE (NG101) guideline, the Society for Integrative Oncology (SIO) guideline, the ONS guideline, and the ACS-ASCO guideline (26, 28, 30, 42, 45). The two SEOM guidelines were rated unsatisfactory quality overall (27, 37), while the remaining guidelines (50%) were rated as moderate quality (recommended with modifications).

The AGREE II domain scores for each guideline varied **(** **Table 3** **)**. The results indicated that the “scope and purpose” domain had the highest mean scores (mean: 84.61%; range: 69.4% -100%), whereas the “applicability” domain had the lowest mean scores (mean: 51.04%; range: 12.5% - 91.7%). More heterogeneous scores were demonstrated in the “stakeholder involvement” domain (mean: 71.43%; range: 36.1% - 100%), “clarity and presentation” domain (mean: 66.15%; range: 31.9% - 97.2%), “rigour of development” domain (mean: 62.32%; range: 18.2% - 92.7%), and “editorial independence” domain (mean: 72.19%; range 33.3% - 91.7%).

**Table 3:** Scores of the domains and overall assessment of the guidelines according to the AGREE II instrument.

Title of Guideline	Domains (%)							Overall Quality	Degree of Recommendation	ICC
	Scope and Purpose	Stakeholder Involvement	Rigor of Development	Clarity and Presentation	Applicability	Editorial Independence	Average			
NICE (CG81) (42)	100	100	92.2	88.9	91.7	89.6	93.73	High	R	0.995
NICE (NG101) (28)	100	100	92.7	88.9	91.7	89.6	93.82	High	R	0.993
Barnadas, Algara (37)	70.8	25.0	50.0	31.9	12.5	70.8	43.50	Low	NR	0.989
St. Gallen International Consensus (38)	69.4	61.1	19.8	38.8	19.7	62.5	45.22	Moderate	RM	0.980
ESMO (EBC) 2019 (39)	73.6	66.7	44.8	41.7	45.8	66.7	56.55	Moderate	RM	0.970
ESO-ESMO (ABC5) 2020 (40)	87.5	70.8	42.7	79.2	55.2	68.8	67.37	Moderate	RM	0.956
ESO-ESMO (BCY4) 2020 (43)	83.3	72.2	63.5	69.4	53.1	68.8	68.38	Moderate	RM	0.973
APTA guideline (41)	95.8	68.1	79.2	81.9	30.2	68.8	70.67	Moderate	RM	0.993
SEOM 2018 (27)	69.4	36.1	18.2	47.2	12.5	33.3	36.12	Low	NR	0.986
SIO guideline (30)	97.2	76.4	89.6	97.2	77.1	91.7	88.20	High	R	0.983
ONS guideline (26)	88.9	88.9	77.1	83.3	77.1	91.7	84.50	High	R	0.991
Reese, Weis (44)	83.3	83.3	53.1	44.4	49.0	66.7	63.30	Moderate	RM	0.985
ACS-ASCO guideline (45)	93.1	93.1	98.0	87.5	83.3	91.7	91.12	High	R	0.992
Sisler, Chaput (29)	72.2	58.3	51.6	45.8	15.6	50.0	48.92	Moderate	RM	0.994
**Average**	84.61	71.43	62.32	66.15	51.04	72.19	67.96	–	–	0.980

ICC, intraclass correlation coefficient; NICE, National Institute for Health and Care Excellence; R, recommended; NR, not recommended; RM, recommended with modifications; ESMO, European Society for Medical Oncology; EBC, early breast cancer; ESO, European School of Oncology; ABC, advanced breast cancer; APTA, American Physical Therapy Association; SEOM, bSociodad Española de Oncolgía Médica; SIO, Society of Interventional Oncology; ONS, Oncology Nursing Society; ACS, American Cancer Society; ASCO, American Society of Clinical Oncology.

### 3.3 Summary of the Self-Managed Non-Pharmacological Interventions

The 14 included CPGs recommended a number of self-managed non-pharmacological interventions for breast cancer survivors. Details of the non-pharmacological interventions recommended by the included guidelines are summarised in **Table 4**.

**Table 4:** Non-pharmacological interventions and therapies recommended by the included clinical practice guidelines.

Clinical Outcomes	Recommended Non-pharmacological Interventions/Therapies	Guidelines	Grading System Used
**Anxiety/depression/distress**	**Meditation** **Form:** MBSR **Duration/Frequency:** NR **LoE/GoR:** I/B (generally recommended) **SoE:** NR	ESO-ESMO (ABC5) 2020	Infectious Diseases Society of America–United States Public Health Service Grading System
	**Hypnosis** **Form:** NR **Duration/Frequency:** NR **LoE/GoR:** I/B (generally recommended) **SoE:** NR	ESO-ESMO (ABC5) 2020	Infectious Diseases Society of America–United States Public Health Service Grading System
	**Yoga** **Form:** NR **Duration/Frequency:** NR **LoE/GoR:** I/B (generally recommended) **SoE:** NR	ESO-ESMO (ABC5) 2020	Infectious Diseases Society of America–United States Public Health Service Grading System
	**Meditation** **Form:** “MBSR Program/Mindful Movement Program/brain wave vibration meditation/Tibetan sound meditation/cognitively based compassion training/Transcendental Meditation” (Greenlee et al., 2017, p. 205) **Duration/Frequency:** NR **LoE/GoR:** A/recommended **SoE:** 10 RCTs and several systematic reviews and meta-analyses	SIO 2017	Modified version of the US Preventive Services Task Force Grading System
	**Music therapy** **Form:** Passive or active music therapy **Duration/Frequency:** NR **LoE/GoR:** B/recommended **SoE:** 5 RCTs and 2 systematic reviews and meta-analysis	SIO 2017	Modified version of the US Preventive Services Task Force Grading System
	**Stress management–for anxiety** **Form:** Self-administered/cognitive-behavioural stress-management **Duration/Frequency:** NR **LoE/GoR:** B/recommended **SoE:** 4 RCTs and 2 systematic reviews	SIO 2017	Modified version of the US Preventive Services Task Force Grading System
	**Yoga** **Form:** Lyengar/Patanjali’s/Pranayama **Duration/Frequency:** NR **LoE/GoR:** B/recommended **SoE:** 15 RCTs and several systematic reviews and meta-analyses	SIO 2017	Modified version of the US Preventive Services Task Force Grading System
	**Relaxation-for depression** **Form:** PMR/guided imagery/visualisation techniques/autogenic training **Duration/Frequency:** NR **LoE/GoR:** A/recommended **SoE:** 6 RCTs and several systematic reviews and meta-analyses	SIO 2017	Modified version of the US Preventive Services Task Force Grading System
	**Massage** **Form:** “classic massage (rhythmic stroking, kneading, and acupressure at select areas on the body)” (Greenlee et al., 2017, p. 212) **Duration/Frequency:** 30-minute massages biweekly, for total of 5 weeks or one time, 40 minutes **LoE/GoR:** B/recommended **SoE:** 6 RCTs and several systematic reviews and meta-analyses	SIO 2017	Modified version of the US Preventive Services Task Force grading system
**Chemotherapy-induced nausea and vomiting**	**Acupressure** **Form:** Self-acupressure **Duration/Frequency:** NR **LoE/GoR:** B/recommended **SoE:** 3 RCTs	SIO 2017	Modified version of the US Preventive Services Task Force Grading System
	**Relaxation** **Form:** NR **Duration/Frequency:** NR **LoE/GoR:** C/considered **SoE:** 2 trials	SIO 2017	Modified version of the US Preventive Services Task Force Grading System
**Fatigue**	**Exercise programme** **Form:** NR **Duration/Frequency:** NR **LoE/GoR:** NR/recommended **SoE:** High-quality systematic review and meta-analysis	NICE 2017	SIGN criteria
	**Exercise** **Form:** NR **Duration/Frequency: “**equivalent to 3-5 hours of moderate walking per week” (Cardoso et al., 2020, p. 1643) **LoE/GoR:** I/A (strongly recommended) SoE: NR	ESO-ESMO (ABC5) 2020	Infectious Diseases Society of America–United States Public Health Service Grading System
	**Meditation** **Form:** MBSR **Duration/Frequency:** NR **LoE/GoR:** I/B (generally recommended) **SoE:** NR	ESO-ESMO (ABC5) 2020	Infectious Diseases Society of America–United States Public Health Service Grading System
	**Hypnosis** **Form:** NR **Duration/Frequency:** NR **LoE/GoR:** I/B (generally recommended) **SoE:** NR	ESO-ESMO (ABC5) 2020	Infectious Diseases Society of America–United States Public Health Service Grading System
	**Yoga** **Form:** NR **Duration/Frequency:** NR **LoE/GoR:** I/B (generally recommended) **SoE:** NR	ESO-ESMO (ABC5) 2020	Infectious Diseases Society of America–United States Public Health Service Grading System
	**Physical activity** **Form:** NR **Duration/Frequency:** NR **LoE/GoR:** I/recommended **SoE:** Several RCTs	ACS-ASCO 2016	Not classified
	**Physical activity** **Form:** NR **Duration/Frequency:** NR **LoE/GoR:** I/recommended **SoE:** NR	Sisler et al. (29)	Not classified
	**Hypnosis** **Form:** NR **Duration/Frequency:** NR **LoE/GoR:** C/considered **SoE:** 2 trials	SIO 2017	Modified version of the US Preventive Services Task Force Grading System
	**Yoga** **Form:** NR **Duration/Frequency:** NR **LoE/GoR:** C/considered **SoE:** 3 trials	SIO 2017	Modified version of the US Preventive Services Task Force Grading System
	**Yoga** **Form:** NR **Duration/Frequency:** NR **LoE/GoR: I**/suggested **SoE:** NR	Sisler et al. (29)	Not specified
**Lymphedema**	**Physiotherapy** **Form:** NR **Duration/Frequency:** NR **LoE/GoR:** I/A (strongly recommended) **SoE:** Meta-analyses of RCTs	ESMO (EBC)	Infectious Diseases Society of America–United States Public Health Service Grading System
	**Physiotherapy** **Form:** NR **Duration/Frequency:** NR **LoE/GoR:** 0/recommended **SoE:** NR	ACS-ASCO 2016	Not classified
	**Physiotherapy** **Form:** Progressive resistance training & aerobic exercise programme **Duration/Frequency:** Aerobic exercise program: **“**pole walking for 30-60 minutes, 3-5 times weekly for 8 weeks; proximal to distal exercise for 45 minutes while integrating self-massage and diaphragmatic breathing and weekly in a 1.2-m pool at 32°C-33°C.” (Davies et al., 2020, p. 1174) **LoE/GoR:** I-II/A (strongly recommended) **SoE:** RCTs	Davies et al. (41)	Oxford Centre for Evidence-Based Medicine–levels of evidence
	**Weight management–risk of lymphedema** **Form:** NR **Duration/Frequency:** NR **LoE/GoR:** 0/recommended **SoE:** NR	ACS-ASCO 2016	Not classified
	**Weight management–risk of lymphedema** **Form:** NR **Duration/Frequency:** NR **LoE/GoR: III**/NR **SoE:** NR	Sisler et al. (29)	Not classified
**Pain**	**Healing touch–after chemotherapy** **Form:** NR **Duration/Frequency:** NR **LoE/GoR:** C/considered **SoE:** A single, large trial	SIO 2017	Modified version of the US Preventive Services Task Force Grading System
	**Music therapy–after surgery** **Form:** NR **Duration/Frequency:** NR **LoE/GoR:** C/considered **SoE:** 2 trials	SIO 2017	Modified version of the US Preventive Services Task Force Grading System
	**Hypnosis–after surgery** **Form:** NR **Duration/Frequency:** NR **LoE/GoR:** C/considered **SoE:** 2 trials	SIO 2017	Modified version of the US Preventive Services Task Force Grading System
	**Physical activity** **Form:** NR **Duration/Frequency:** NR **LoE/GoR:** I/recommended **SoE:** NR	Sisler et al. (29)	Not classified
	**Physical activity** **Form:** NR **Duration/Frequency:** NR **LoE/GoR:** I/recommended **SoE:** NR	Sisler et al. (29)	Not classified
	**Physical activity** **Form:** NR **Duration/Frequency:** NR **LoE/GoR:** I/recommended **SoE:** Many RCTs, meta-analyses of RCTs	ACS-ASCO 2016	Not classified
**Neuropathy**	**Physical activity** **Form:** NR **Duration/Frequency:** NR **LoE/GoR:** IA/recommended **SoE:** NR	ACS-ASCO 2016	Not classified
**Quality of life**	**Regular exercise/sport** **Form:** NR **Duration/Frequency: “**equivalent to 3-5 hours of moderate walking per week” (Cardoso et al., 2020, p. 1643) **LoE/GoR:** I/B (generally recommended) **SoE:** NR	ESO-ESMO (ABC5) 2020	Infectious Diseases Society of America–United States Public Health Service Grading System
	**Meditation** **Form:** MBSR **Duration/Frequency:** NR **LoE/GoR:** I/B (generally recommended) **SoE:** NR	ESO-ESMO (ABC5) 2020	Infectious Diseases Society of America–United States Public Health Service Grading System
	**Hypnosis** **Form:** NR **Duration/Frequency:** NR **LoE/GoR:** I/B (generally recommended) **SoE:** NR	ESO-ESMO (ABC5) 2020	Infectious Diseases Society of America–United States Public Health Service Grading System
	**Yoga** **Form:** NR **Duration/Frequency:** NR **LoE/GoR:** I/B (generally recommended) **SoE:** NR	ESO-ESMO (ABC5) 2020	Infectious Diseases Society of America–United States Public Health Service Grading System
	**Meditation** **Form:** “MBSR program/Mindful Movement Program/brain wave vibration meditation/Tibetan sound meditation/cognitively based compassion training/Transcendental Meditation” (Greenlee et al., 2017, p. 215) **Duration/Frequency:** NR **LoE/GoR:** A/recommended **SoE:** 7 RCTs and several systematic reviews and meta-analyses	SIO 2017	Modified version of the US Preventive Services Task Force Grading System
	**Yoga** **Form:** Lyengar/Patanjali’s/Pranayama/integrated yoga **Duration/Frequency:** NR **LoE/GoR:** B/recommended **SoE:** 12 RCTs and several systematic reviews and meta-analyses	SIO 2017	Modified version of the US Preventive Services Task Force Grading System
	**Qigong** **Form:** NR **Duration/Frequency:** NR **LoE/GoR:** NR/C (considered) **SoE:** NR	SIO 2017	Modified version of the US Preventive Services Task Force Grading System
	**Stress management** **Form:** NR **Duration/Frequency:** NR **LoE/GoR:** NR/C (considered) **SoE:** NR	SIO 2017	Modified version of the US Preventive Services Task Force Grading System
**Sleep disturbance**	**Yoga** **Form:** NR **Duration/Frequency:** NR **LoE/GoR:** C/considered **SoE:** 5 trials	SIO 2017	Modified version of the US Preventive Services Task Force Grading System
	**Relaxation training** **Form**: NR **Duration/Frequency:** NR **LoE/GoR:** NR **SoE:** NR	Reese et al. (44)	No grading system
**Vasomotor/hot flashes**	**Physical activity** **Form:** Exercise/yoga **Duration/Frequency:** 8 to 12 weeks, and follow-up 3 to 6 months varied **LoE/GoR: L**ow/conditional **SoE:** 3 trials	ONS 2020	GRADE
**Risk of recurrence**	**Physical activity** **Form:** NR **Duration/Frequency:** NR **LoE/GoR:** NR **SoE:** Low-quality evidence from 2 cohort studies	NICE 2018	SIGN criteria
	**Weight management** **Form:** NR **Duration/Frequency:** NR **LoE/GoR:** NR **SoE:** Moderate-quality evidence from 1 RCT	NICE 2018	SIGN criteria
	**Lifestyle change** **Form:** NR **Duration/Frequency:** NR **LoE/GoR:** NR **SoE:** NR	St. Gallen 2019	Not classified
	**Physical exercise** **Form:** NR **Duration/Frequency:** NR **LoE/GoR:** II/A (strongly recommended) **SoE:** NR	SEOM 2018	Infectious Diseases Society of America–United States Public Health Service Grading System
	**Weight management** **Form:** NR **Duration/Frequency:** NR **LoE/GoR:** II/A (strongly recommended) **SoE:** NR	SEOM 2018	Infectious Diseases Society of America–United States Public Health Service Grading System

MBSR, mindfulness-based stress reduction; NR, not reported; LoE, level of evidence; GoR, grade of recommendation; SoE, strength of evidence; ESO, European School of Oncology; ESMO, European Society for Medical Oncology; ABC, advanced breast cancer; SIO, Society of Interventional Oncology; US, United States; RCT, randomised controlled trial; NICE, National Institute for Health and Care Excellence; SIGN, Scottish Intercollegiate Guidelines Network; ACS, American Cancer Society; ASCO, American Society of Clinical Oncology; ONS, Oncology Nursing Society; GRADE, Grading of Recommendations Assessment, Development, and Evaluation; SEOM, Sociodad Española de Oncolgía Médica.

#### 3.3.1 Anxiety, Depression, and Distress

Two CPGs recommended the use of meditation, in particular, mindfulness-based stress reduction (MBSR) and yoga, to alleviate the symptoms of anxiety, depression, and distress (30, 40). For example, one CPG mentioned a published randomised controlled trial (RCT) using the Mindful Movement Program, including mindful moving, body parts exploration, deliberate and active movement, and group discussion for anxiety/stress reduction (30). In addition, massage therapy (kneading, rhythmic stroking, and acupressure), passive music therapy, and hypnosis were also recommended by the two CPGs (30, 40). In terms of duration and frequency of massage, 30-minute classic massage therapy performed biweekly for five weeks was recommended by five out of the six RCTs included in (30). The two guidelines did not describe the specific elements and duration of music and hypnosis therapy (30, 40). One CPG contemplated specifically in its recommendations the adoption of stress management (self-administered or cognitive-behavioural stress-management) for anxiety reduction, and relaxation (progressive muscle relaxation, guided imagery, visualisation techniques, and autogenic training) for depression alleviation (30). There was a similarity in terms of the SoE, and all evidence was from RCTs and/or systematic reviews.

#### 3.3.2 Fatigue

There was a consensus regarding the effectiveness of physical activity/exercise in reducing fatigue among breast cancer survivors, with five analysed CPGs highly to moderately recommendable (29, 40, 42, 44, 45). In particular, one guideline with a high LoE (Level I) recommended physical sports/exercise which equalled three to five hours of moderate walking every week. Yoga was considered likely to improve fatigue by three CPGs (29, 30, 40). Similarly, hypnosis therapy was recommended/considered by two CPGs to improve the symptom of fatigue (30, 40), and MBSR was recommended by one of the CPGs, with a high LoE (Level I) (40); however, neither of the included CPGs provided sufficient details about the doses of their interventions. It was highlighted that some of the CPGs that recommended interventions for reducing the symptom of fatigue did not report an LoE (29, 30, 40).

#### 3.3.3 Pain

Two CPGs with a high LoE (Level I), recommended applying physical activity to reduce pain (29, 45). Nevertheless, these two guidelines did not specify the forms, duration, and frequency of the physical activities. Music therapy (after surgery) and hypnosis (after surgery) was considered for pain relief in one of the CPGs (30); however, the GoR presented by this CPG was Grade C (low).

#### 3.3.4 Breast Cancer-Related Lymphedema

Three CPGs recommended physiotherapy to reduce breast cancer-related lymphedema (BCRL) (39, 41, 45), while only one guideline specified progressive resistance training (PRT) as a safe practice at least one month after surgery (41). For example, Davies et al. (41) mentioned one RCT that applied supervised PRT to women with breast cancer for the first 20 weeks, followed by 30 weeks of self-managed resistance exercises (41), but the RCT did not provide any evidence that PRT prevented lymphedema, rather, the results validated the safety of PRT. Davies, Levenhagen (41) also recommended that aerobic exercise should be offered to women who have BCRL (Stage 0-III), with a high or medium LoE. For example, patients performed aquatic exercises in a proximal-to-distal sequence for 45 minutes in a 1.2 meter pool at 32 to 33 degrees Celsius while integrating self-massage and diaphragmatic breathing, and the frequency was once every week (41). Although this protocol demonstrated a minor reduction in BCRL, a long-term effect was not shown at 12 weeks follow-up. In addition, two CPGs suggested weight management to reduce the risk of BCRL, but the specific amount of weight a patient should aim to lose was not reported (29, 45). It is noteworthy that the LoE found in the two CPGs was low or very low (Level III or below).

#### 3.3.5 Other Symptoms

Only one of the included CPGs considered the use of self-acupressure and relaxation to a moderate degree (Grade B and Grade C) to control chemotherapy-induced nausea and vomiting (CINV) in addition to drug treatment (30). The evidence from one review and one RCT demonstrated the effectiveness of Neiguan acupoint (P6) acupressure using a wristband on both arms to alleviate CINV (46, 47). Only one CPG recommended the application of physical activity to lessen the neuropathies caused by the breast cancer itself or the surgery/chemotherapy treatments received, and the LoE was Level IA (high) (45). Nevertheless, the form, duration, and frequency of physical activity was not reported. With regards to sleep disturbance, one CPG reported that yoga should be considered to improve symptoms (30). In addition, another CPG, without reporting the LoE and GoR, suggested that relaxation training should be offered (44). One CPG with a low LoE recommended physical activity to alleviate vasomotor/hot flashes presented by breast cancer patients, such as exercise or yoga (26); in addition, this same guideline also recommended that hypnosis and relaxation therapy might be two promising approaches to reducing vasomotor/hot flashes based on limited research evidence. Further, none of the included guidelines reported any non-pharmacological interventions for bone health management.

#### 3.3.6 Quality of Life

Regarding the improvement of QoL among breast cancer survivors, regular exercise or sports was recommended, with a high LoE (Level I), by one CPG (40), and the duration and frequency was equivalent to three to five hours of moderate walking every week. Meditation, in particular MBSR, and yoga (Lyengar, Patanjali’s, Pranayama, or integrated yoga programme) were recommended as approaches to improving QoL by two CPGs (30, 40), with the SoE from RCTs, systematic reviews, and meta-analyses. In addition, one of the CPGs recommended the application of hypnosis to improve QoL, with a high LoE (Level I) (40), while the other CPG recommended qigong to enhance QoL as well as stress management based on the evidence of seven trials (30); nevertheless, there were conflicting results reported in the trials. However, the two CPGs SoE and the sample sizes were fairly small.

#### 3.3.7 Risk of Recurrence

Two CPGs contemplated physical activity and exercise to reduce the risk of recurrence of breast cancer; however, both CPGs presented either a medium or low LoE (27, 28). The reduction of the likelihood of recurrence when practicing the weight management approach produced controversy in different CPGs. Three guidelines, with a medium LoE, recommended weight loss to reduce the risk of recurrence (27, 28, 37), while one guideline suggested that weight loss did not affect the risk of recurrence of breast cancer (38); without providing the LoE and GoR.

#### 3.3.8 Health Promotion

Five self-managed non-pharmacological approaches were recommended for health promotion in breast cancer survivors, including weight management, physical activity, nutrition, alcohol limitation, and smoking cessation **(** **Table 5** **)**. Weight management, in the form of limited high-calorie beverage and food, was recommended by one CPG (45). There was clear consensus regarding the benefits of physical activity for breast cancer survivors, with five CPGs recommending it (29, 37, 39, 44, 45). The duration and frequency were 75 minutes of vigorous or 150 minutes of moderate aerobic exercise every week. The LoE presented by these five CPGs was high (Level I). A balanced diet, including high amounts of vegetables, fresh fruit, and legumes, as well as reduced processed food and red meat and low amounts of saturated fats, was commonly recommended by four CPGs (29, 37, 39, 45), with a high LoE (Level I). Three CPGs suggested limited alcohol consumption of 1 unit or 20g per day (29, 37, 45); however, the LoE reported by those CPGs was low. Smoking cessation was recommended by two CPGs (29, 45), with a high LoE (Level I).

**Table 5:** Health promotion for breast cancer survivors per the included clinical practice guidelines.

	Recommendations	Guidelines	Grading System Used
**Weight management**	**Form: L**imited high-calorie foods and beverages intake **Duration/Frequency:** NR **LoE/GoR:** IA, III/recommended **SoE:** NR	ACS-ASCO 2016	Not classified
**Physical activity**	**Form:** Aerobic exercise **Duration/Frequency:** “150 min of moderate or 75 min of vigorous aerobic exercise per wk” (Runowicz et al., 2016, p. 64) **LoE/GoR:** I, IA/recommended **SoE:** NR **Form:** Strength training exercises **Duration/Frequency:** at least 2d per wk **LoE/GoR:** IA/recommended **SoE:** NR	ACS-ASCO 2016	Not classified
	**Form:** Vigorous physical activity **Duration/Frequency:** 150 min per week **LoE/GoR:** NR **SoE:** systematic review	Barnadas et al. (37)	No grading system
	**Form:** Strength training exercises and vigorous physical activity **Duration/Frequency:** “physical activity: 150 minutes of moderate or 75 minutes of vigorous physical activity per week; strength training exercises: 2 d/wk” (Sisler et al., 2016, p. 809) **LoE/GoR:** I/recommended **SoE:** systematic review	Sisler et al. (29)	Not classified
	**Regular exercise** **Form:** NR **Duration/Frequency:** NR **LoE/GoR:** II/B (general recommended) **SoE:** NR	ESMO (EBC)	Infectious Diseases Society of America–United States Public Health Service Grading System
	**Physical exercise**	Reese et al. (44)	No grading system
**Nutrition**	**A balanced diet**	Reese et al. (44)	No grading system
	**Form:** nutritional counselling **Duration/Frequency:** NR **LoE/GoR:** III/B (generally recommended) **SoE:** NR	ESMO (EBC)	Infectious Diseases Society of America–United States Public Health Service Grading System
	**Form:** “high in vegetables, fruits, whole grains, and legumes; low in saturated fats” (Runowicz et al., 2016, p. 65) **Duration/Frequency:** NR **LoE/GoR:** IA, III**/**recommended **SoE:** NR	ACS-ASCO 2016	Not classified
	**Form:** “low-fat diet; high in fresh fruits, vegetables, and legumes (at least two pieces of fruit per day); lower their intake of red meat (to 1-2 times per week) and processed meats; increase consumption of blue fish, olive oil use and consume dairy products” (Barnadas et al., 2018, p. 691)	Barnadas et al. (37)	No grading system
	**Form**: “high in vegetables, fruits, whole grains, and legumes; low in saturated fats and limited in processed and red meats” (Sisler et al., 2016, p. 809) **Duration/Frequency:** NR **LoE/GoR:** I**/**recommended **SoE:** NR	Sisler et al. (29)	Not classified
**Alcohol limitation**	**Form:** “abstain from drinking more than 20g of alcohol per day” (Barnadas et al., 2018, p. 691) **LoE/GoR:** NR **SoE:** NR	Barnadas et al. (37)	No grading system
	**Limited alcohol consumption** **LoE/GoR:** 0/NR **SoE:** NR	ACS-ASCO 2016	Not classified
	**Limit alcohol to 1 unit/day** **LoE/GoR:** III/recommended **SoE:** NR	Sisler et al. (29)	Not classified
**Smoking cessation**	**Avoid smoking** **LoE/GoR:** I/NR **SoE:** NR	ACS-ASCO 2016	Not classified
	**Smoking cessation** **LoE/GoR:** I/recommended **SoE:** NR	Sisler et al. (29)	Not classified

NR, not reported; LoE, level of evidence; GoR, grade of recommendation; SoE, stremgth of evidence; ACS, American Cancer Society; ASCO, American Society of Clinical Oncology; ESMO, European Society for Medical Oncology EBC, early breast cancer.

## 4 Discussion

This review systematically appraised the quality of 14 published CPGs and further clarified and synthesised the evidence bases regarding self-managed non-pharmacological interventions for breast cancer survivors. The summarised evidence can be utilised by healthcare professionals to guide breast cancer survivors in applying self-managed strategies to manage their long-term symptoms.

### 4.1 Quality of the Clinical Practice Guidelines

This review highlighted that the quality of the included CPGs had much room for improvement, which was particularly obvious in the “applicability” and “rigour of development” domains in the AGREE II tool. It was reported that the median scores of the “scope and purpose” domain for most of the CPGs were > 70%, indicating that most of the included CPGs had clear purposes for guideline development. In contrast, the “applicability” domain had the lowest median scores, suggesting that the facilitation of and the barriers to the CPGs’ implementation were not appropriately addressed. To facilitate the implementation of the CPGs, some barrier analysis and/or pilot studies should be conducted to identify the barriers to their implementation (48). In addition, engaging end-users and other stakeholders (e.g., patients, patient advocacy, policymakers, etc.) in the CPGs’ development of non-pharmacological interventions for cancer survivors could help to enhance the incorporation of CPGs and to ensure that the interventions and therapies are sustainable and clinically feasible (49).

Regarding the “rigour of development” domain, this review found that the majority of the CPGs did not use a systematic approach in the formulation of the guidelines, indicating a dearth of systematic review methodologies for the synthesis of evidence in the analysed CPGs. A systematic review approach can be used to identify relevant evidence and to sufficiently describe the methodologies for developing the recommendations. In addition, the CPGs update frequency varied, with only approximately half of the guidelines indicating schedules for updating their guidelines. Scientific evidence will have advanced much quicker than the scheduled updates of the guidelines; hence, more timely updates underpinned by the latest evidence would enhance the implementation and acceptance of these CPGs (50).

### 4.2 Content Analysis of the Clinical Practice Guidelines

Although many CPGs have been developed for breast cancer diagnosis and treatment, few CPGs have addressed non-pharmacological interventions for breast cancer survivors (45). Due to the significantly heterogeneous LoE presented in the included CPGs, the majority of the evidence bases was not adequate to warrant a strong recommendation for the effectiveness of the self-managed non-pharmacological interventions in various clinical outcomes. Physical activity, particularly in the form of regular physical exercise, was the only core self-managed non-pharmacological intervention for psychological and physical symptom management recommended in all the included CPGs. However, the form, duration, and frequency of the different physical activities were not adequately described. A recent pilot study showed that a physical exercise rehabilitation program was effective in reducing breast cancer fatigue at both post-intervention and follow-up time points (11). This physical exercise rehabilitative protocol incorporated 10 min of warm-up, followed by aerobic exercise and strength training (40 min), and then cool-down (10 min), twice a week in a 4-week period of time. Future CPGs might consider providing more details of the recommended interventions, particularly the recommended duration, frequency, delivery method, and sessions, etc.

Similarly, some evidence bases supported the application of other self-managed non-pharmacological interventions, such as meditation, relaxation, stress management, music therapy, yoga, massage, and acupressure, for breast cancer survivors. Nevertheless, a clear understanding of which specific modality of each intervention was effective and acceptable to breast cancer survivors was lacking. Rather, most of the recommendations, such as yoga, meditation, relaxation, etc., were largely deemed as possibly effective self-management strategies for breast cancer survivors given the limitations in relation to low LoE and inconsistency in the GoR of the evidence bases. Some interventions, such as qigong and stress management for reducing clinical symptoms were demonstrated as effective in trial settings only; however, few evidence bases of these interventions were successfully translated to a wide range of populations. The review highlighted that the effectiveness of self-managed non-pharmacological interventions in bone health management among breast cancer patients receiving aromatase inhibitors was under-represented in the included CPGs. One pilot randomized study reported that physical exercises together with whole-body vibration demonstrated a significant effect on pain, muscle functioning, strength, and QoL in breast cancer survivors with aromatase inhibitor-induced musculoskeletal symptoms (51). More evidence base supporting the effect of non-pharmacological strategies on bone health in breast cancer survivors receiving aromatase inhibitors are warranted.

Current evidence bases of the included CPGs supported the efficacy of physical activity, weight management, nutrition, limited alcohol consumption, and smoking cessation in improving breast cancer survivorship outcomes in the “health promotion” domain. Nevertheless, the quality of the evidence was inconsistent or poor for particular topics within the “health promotion” domain, such as losing weight and a reduction/cessation of alcohol and tobacco consumption. For example, although weight loss was recommended by some guidelines, there were some ambiguities in terms of the specific amount of weight that breast cancer survivors should lose. Teras, Patel (52) showed that in women aged 50 years and over, women with sustained weight loss (at least 2 kg) had a lower risk of breast cancer, and those who lost at least 9 kg had the lowest risk, compared with women with a stable weight.

### 4.3 Implications for Future Research and Clinical Practice

Clinicians might consider using the results of this review as a potential guide in choosing high-quality guidelines to inform the self-managed non-pharmacological interventions that could be recommended to breast cancer survivors. For example, when designing an intervention programme to alleviate fatigue in breast cancer survivors, physical activity/exercise with a duration and frequency equal to three to five hours of moderate walking per week could be considered and adjusted based on the breast cancer survivors’ actual condition. In addition, the results of the quality appraisal using the AGREE II assessment tool can help guideline developers to determine which domain needs to be further strengthened. In particular, the domain of “applicability” should be addressed adequately. To enhance overall quality, the development of CPGs may consider taking the AGREE II’s “rigor of development” domain into account.

The review findings also provided some directions for further research. This study emphasised the inconsistency of evidence bases for some of the recommendations in the analysed CPGs. It is necessary to conduct more large-scale and rigorously designed RCTs to consolidate the evidence bases and further define the effectiveness of various non-pharmacological interventions whose evidence bases are not adequately strengthened or are contradictory, such as the effectiveness of qigong and stress management on QoL in breast cancer survivors. In addition, given the current person-centred care approach, considering the views of breast cancer survivors as the end-users on the flexibility and usefulness of the CPGs would be crucial to ensure that the self-managed non-pharmacological interventions are properly designed to meet their needs. The review findings also encourage future research to explore strategies to support the best translation of research evidence to clinical practice, such as strategies to support clinicians in adhering to guidelines to provide evidence-based treatment and care (53).

### 4.4 Study Limitations

This review has some limitations. Only guidelines published in English were included, therefore non-English-language guidelines might have been missed. One presumed limitation of this review could be the subjective process of assessment, which might have had an impact on the rating of the items, the global guideline appraisal, and the degree of the recommendations. However, in this review, the ICC was > 98%, indicating excellent agreement among the four experienced assessors.

## 5 Conclusion

Physical activity/exercise, meditation, hypnosis, yoga, music therapy, stress management, relaxation, massage and acupressure were frequently recommended by CPGs as promising self-managed non-pharmacological interventions for breast cancer survivors, of which physical activity/exercise was the most commonly recommended intervention for the management of psychological and physical symptoms. However, this study indicated that recommendations for the self-managed non-pharmacological interventions were varied and limited among the included CPGs, and some were based on medium- and low-quality research evidence. More rigorous methods are required to develop high-quality CPGs to guide clinicians in offering high-quality and tailored breast cancer survivorship care.

## Author Contributions

J-YT: study conceptualisation and design, methodology, and manuscript revision; JZ: study design, methodology, manuscript drafting and revision; TW: study conceptualisation and design, methodology, review, and editing; H-JZ: data analysis and manuscript revision; IZ: study design and manuscript revision; X-LL: study design, methodology, review, and editing. All authors contributed to the article and approved the submitted version.

## Funding

This study was support by the COVID- 19 Supplementary Funding Pool Scheme and the Institute of Advanced Studies (IAS) Rainmaker Start-Up Grant at Charles Darwin University.

## Conflict of Interest

The authors declare that the research was conducted in the absence of any commercial or financial relationships that could be construed as a potential conflict of interest.

## Publisher’s Note

All claims expressed in this article are solely those of the authors and do not necessarily represent those of their affiliated organizations, or those of the publisher, the editors and the reviewers. Any product that may be evaluated in this article, or claim that may be made by its manufacturer, is not guaranteed or endorsed by the publisher.
